# The Transformative Impact of Community-Led Monitoring in the South African Health System: A Comprehensive Analysis

**DOI:** 10.3389/ijph.2024.1606591

**Published:** 2024-02-14

**Authors:** Ndumiso Tshuma, Daniel Ngbede Elakpa, Clinton Moyo, Melikhaya Soboyisi, Sehlule Moyo, Sihlobosenkosi Mpofu, Martha Chadyiwa, Mokgadi Malahlela, Caroline Tiba, David Mnkandla, Tshepo M. Ndhlovu, Tsenolo Moruthoane, David D. Mphuthi, Oliver Mtapuri

**Affiliations:** ^1^ The Best Health Solutions, Johannesburg, South Africa; ^2^Center for Health Policy, School of Public Health, Faculty of Health Sciences, University of the Witwatersrand, Johannesburg, South Africa; ^3^ Networking HIV and AIDS Community of Southern Africa (NACOSA), Gauteng, South Africa; ^4^ Department of Environmental Health, Faculty of Health Sciences, University of Johannesburg, Johannesburg, South Africa; ^5^ Rotanganeza Community Care, West Rand, South Africa; ^6^ Office of the Premier, Free State, South Africa; ^7^ College of Human Sciences, University of South Africa, Pretoria, South Africa; ^8^ School of Built Environment and Development Studies, College of Humanities, University of KwaZulu-Natal, Durban, South Africa

**Keywords:** community-led monitoring, data value chain, human-centered design, affinity diagramming, stakeholders

## Abstract

**Objectives:** Community-led monitoring (CLM) is an emerging approach that empowers local communities to actively participate in data collection and decision-making processes within the health system. The research aimed to explore stakeholder perceptions of CLM data and establish a CLM Data Value Chain, covering data collection and its impact.

**Methods:** Qualitative data were collected from stakeholders engaged in health programs in South Africa. Data analysis involved a collaborative workshop that integrated elements of affinity diagramming, thematic analysis, and the systematic coding process outlined in Giorgi’s method. The workshop fostered joint identification, co-creation of knowledge, and collaborative analysis in developing the data value chain.

**Results:** The findings showed that CLM data enabled community-level analysis, fostering program advocacy and local collaboration. It enhanced program redesign, operational efficiency, and rapid response capabilities. Context-specific solutions emerged through the CLM Data Value Chain, promoting sustainable and efficient program implementation.

**Conclusion:** CLM is a powerful tool for improving program implementation, quality, and advocacy in South African healthcare. It strengthens accountability, trust, and transparency by involving local communities in data-driven decision-making. CLM addresses context-specific challenges and tailors interventions to local needs.

## Introduction

Community-led monitoring (CLM) has emerged as a critical accountability mechanism for HIV responses at different levels, spearheaded by local community-led organizations, networks of key populations, and other affected groups in accordance with the UNAIDS guidelines [1–3]. In South Africa, CLM as a Community Systems Strengthening (CSS) programme focuses on strengthening community systems for scaled-up and sustainable community-based responses [4]. The South African CLM approach is adapted from the Ritshidze and other models [2, 4–6]. The model accomplishes work through identification and collection of data, analyses and interpretation data, dissemination of information, advocacy for solutions and monitoring the change [4, 7–9]. This has been instrumental since the onset of the HIV epidemic, playing a vital role in gathering treatment information, securing political support, and funding research [10]. While CLM has achieved significant successes globally, its impact in South Africa has not translated into substantial results. Although it enhances data capacity, analysis, security, and ownership [2, 11, 12], a significant challenge lies in the underutilization of CLM data in the South African context [11, 13]. The increasing availability of CLM data in South Africa emphasizes the importance of going beyond documentation and focusing on result-oriented actions. Recent data collection efforts revealed that 20% of the key population encountered barriers in accessing treatment, including negative experiences with clinical staff, limited safety and privacy, and denied services [14].

CLM has been implemented in countries such as Indonesia, Nepal, Papua New Guinea, Ghana, Mexico, and Bangladesh [15–17]. Across these countries, they learnt that the success of CLM is dependent on community members’ willingness to participate. Therefore, building trust and maintaining open communication with the community is crucial [12, 15, 18–20]. In certain instances, CLM data tends to accumulate without translating into concrete improvements in services, programs, and policies [21]. To address this, decision-making authorities must possess the capacity to understand and effectively utilize CLM data. “CLM data use” entails several essential actions, such as data cleaning, making data accessible, community data analysis, communication and advocacy, action-taking in response to community needs, and enhancement of health service uptake. This process should be continuously tracked to ensure ongoing progress [2, 22, 23]. However, little is known about how communities and stakeholders in the South African health continuum interact with CLM data. In light of this gap, this research will seek to answer the question: what are the key components and stages in the CLM Data Value Chain that contribute to the transformative impact of CLM.

The study aimed will be to comprehensively explore the perceptions of stakeholders engaged in health programs in South Africa regarding the value chain of CLM data usage at the community level. Additionally, our research sought to establish a comprehensive CLM data value chain, encompassing the entire process, starting from data collection and extending to the potential impact of data on decision-making and program improvements.

## Methods

### Study Design

This study is grounded in a theoretical framework that integrates Amedeo Giorgi’s Descriptive Phenomenological Method [24, 25], Human-Centered Design [26–29] and Participatory Action Research [30, 31]. The qualitative phenomenological design was selected to provide a deep exploration of the participants’ experiences in transforming CLM data into actionable information [25]. Drawing from Giorgi’s method and Affinity Diagramming method [32], the study aimed for a systematic process of bracketing, intuiting, analysing, and describing the essential structure of the CLM data handling and processing, preserving the integrity of healthcare worker’s lived experiences.

To ensure the study’s relevance to the stakeholders involved in the CLM program, the design incorporated principles from Human-Centered Design. This approach prioritized understanding stakeholder’s perspectives and experiences [26, 28]. Iterative processes of prototyping, testing, and refining the CLM data value chain based on the feedback from stakeholders were embedded in the research design. Moreover, the study design embraced the ethos of Participatory Action Research (PAR).

### Study Setting

The West Rand District Municipality is located within the western part of the Gauteng Province in South Africa, with its unique healthcare challenges and opportunities, serves as a microcosm for examining the transformative aspects of CLM data in improving health service delivery [33].

### Participant Recruitment

Purposive selection of participants was done based on active involvement in the CLM programme since inception. A total of 23 representatives from CLM implementing CBOs and Department of Health in the West-Rand District in South Africa participated in this study. Verbal consent was sought from each participant, emphasizing the voluntary nature of participation and ensuring confidentiality. Anonymization procedures were implemented to safeguard participant identities.

### Data Collection

Data were collected using semi-structured interviews whose design was informed by Giorgi’s Descriptive Phenomenological Method [25]. Open-ended questions were crafted to capture the essential structures of the participants’ experiences in transforming CLM data. The background and involvement section sought insights into their role and engagement with CLM data transformation. Participants were then invited to articulate their understanding of the essential structures or key elements integral to the transformation of CLM data into actionable information. Further, the interview explored the local impact of CLM data, examining how participants have observed its use in enhancing health service delivery and encouraging specific examples of local changes catalysed by CLM data. Active recording, comprehensive note-taking, and observation of non-verbal cues during interviews were consistent with Giorgi’s emphasis on preserving the nuances of lived experiences.

### Data Analysis and Management

Data analysis involved a collaborative workshop that integrated elements of affinity diagramming, thematic analysis, and the systematic coding process outlined in Giorgi’s method [25]. The workshop fostered joint identification and co-creation of knowledge in developing the data value chain. We used a thematic analysis involving an inductive coding process of the qualitative data into clusters of similar themes, patterns, and relationships between themes, aiming to derive a theoretical explanation CLM data value chain [34]. To ensure rigor and reliability, a team of five authors participated in the coding process, resolving any disagreements through a democratic voting process.

### Ethical Considerations

This study was approved by the University of Johannesburg Faculty of Health Sciences Research Ethics Committee (clearance number: REC-1730-2022). The participants were also informed that their responses would be recorded and subjected to analysis and publication. They were informed about the objectives of the study, their freedom to participate or withdraw from the study at any time if they wished, the confidentiality of information, and the possible risks and benefits of the study.

## Results

A total of 23 stakeholders participated in the study. The gender distribution were 15 females, six males, and two transgender individuals. The CBOs representatives were 18, while five were from the Department of Health in the West-Rand District in South Africa. All participants have been actively involved in the CLM programme since inception in the district.

**FIGURE 1:**
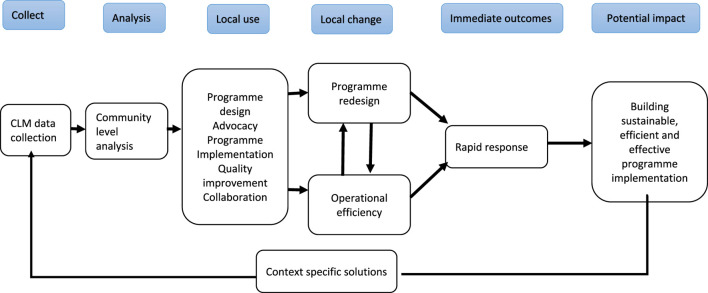
Community led monitoring value chain (West Rand, South Africa. 2023).

The results are presented in accordance with the proposed co-created CLM Data Value Chain framework outlined in Figure 1 below. Meaningful units derived from the thematic analysis were condensed into overarching themes aligned with the categories: 1) data collection, 2) analyse, 3) local use, 4) local change, and 5) immediate outcomes, and 5) potential impacts. This synthesis of findings aimed to provide a structured yet nuanced understanding of stakeholders’ experiences in transforming CLM data. The participants indicated that they were able to collect CLM data using a wide range of qualitative and quantitative methods. Their methods included conducting door-to-door surveys in areas served by clinics, conducting direct observation, conditions of health services received, interviewing staff members in health facilities, and facilitating focus group meetings. Most of these participants recognized the positive attributes of CLM data in bringing positive changes for the communities.

Data sharing between implementing organisations and Department of Health proved instrumental in addressing programme gaps. CLM data were used to generate actionable community driven change. The information generated through individual stories or unique observations brought out important issues that have received little attention and remain unaddressed. Based on the findings of this study, we developed CLM Data Value Chain framework (Figure 1 below). The framework shows a coherent sequence of events commencing with the collection of CLM data, the process unfolds with a subsequent community-level analysis, facilitating a comprehensive understanding of the prevailing challenges and opportunities. This analytical insight, in turn, drives the inception of well-informed program design and prompts proactive advocacy efforts to garner support for the envisioned interventions.

As the program gains momentum, it undergoes rigorous implementation, continuously adapting and refining based on the lessons learned. Notably, a culture of quality improvement is fostered, optimizing the efficacy and impact of the program at the local level. Collaboration among stakeholders becomes a pivotal element, forging strong partnerships to address shared objectives and overcome obstacles collectively. This cohesive approach results in tangible changes and iterative program redesigns, boosting operational efficiency and ensuring that the program remains responsive and adaptable to evolving circumstances. The immediate outcomes are characterized by swift and effective responses to emerging challenges, paving the way for a promising potential impact which is the establishment of sustainable, efficient, and effective program implementation.

In a repetitive pattern, these achievements circle back to the genesis of context-specific solutions, custom-tailoring interventions to the unique needs and nuances of the community. This holistic approach perpetuates the CLM Data Value Chain, promoting continuous data collection as new insights emerge, sustaining the journey towards progressive transformation and data-driven decision-making.

### Community Led Monitoring Value Chain

Below is a diagrammatic illustration of CLM value change and its impact on the community.

### Positive Proponents of CLM Among Stakeholders

#### Collect

Drawing lessons from information collected in clinics, a community implementer noted that CLM data collection helped to identify internal gaps in the healthcare system. She said they managed to address the lack of record keeping for gender based violence (GBV) statistics at some local clinics where records were not being kept for victims of violence.

“*Other clinics don’t have record keeping so we saw a gap in record keeping. Therefore, CLM filled the gap where records such as GBV statistics were not being kept.*”

**CBO representative-1, Data Collector**

#### Analysis

A member of a community-based organization said that data collection by different stakeholders led to data validation. Through CLM data collected and observations they noticed an increase in teenage pregnancy which was an indication of low uptake of contraceptives by teenagers. Subsequently, data were validated by a wider range of stakeholders during community consultative group meetings.

*“Because we looked at teenage pregnancy before COVID and after COVID or during COVID you could see how it (teenage pregnancy numbers went up) shot up and it was clear evidence that contraception services had collapsed. And l think CLM helped all the relevant stakeholders* (stakeholders in community consultative group meetings)...*”*

**CBO representative-2, Data Collector**

One of the Project Managers from a community-based organization indicated that there was a misconception that once you are on ARVs you are cured of HIV. Through the CLM data collection process Data Collectors conducted FGDs and analysed the data so as to establish the driver of this misinformation and misunderstanding.

*Because we were trying to get the people to take their ARVs. So, we got some information that the (ART Roll out) campaign was misunderstood that if people take their ARVs they no longer have HIV (they are cured of HIV) … So, they (CLM data collectors) did FGDs to find out what was understood about the campaign. There was a report generated on how best to roll out and educate the communities.*

**CBO representative-3, Project Manager**

#### Local Use

Data collected through CLM lead to improved program implementation, enhanced advocacy activities, and also facilitated program redesign at a local level. As a result of CLM data collection quality improvements and collaborative activities were also some of the changes identified by the participants.

##### Advocacy

According to an Advocacy Officer, CLM data are integrated into evidence-based advocacy, where information is brought into the attention of relevant decision-makers within the Department of Health. This information gathered and presented to these decision makers is used to hold them accountable in a non-judgemental manner.

*I think the other thing about CLM is that it brings that objectivity, so the information is not necessarily subjective. The issues that are highlighted are advocacy issues … having identified the proper decision makers we are able to make that change by demanding relevant decision makers to act after we have presented our findings to them.*

**CBO representative-4, Advocacy Officer**

Another community-based organizational representative reiterated that CLM qualitative data collected through photo voice was key in influencing decision makers. Photovoice is a participatory research method that combines photography and storytelling. It empowers individuals or communities to communicate their experiences and perspectives on a specific issue or topic. Participants are given cameras or smartphones to take photographs representing their lived experiences, which serve as visual data. Group discussions and reflections are then conducted to analyse common themes and insights. Photovoice projects aim to promote social change, raise awareness, and empower marginalized voices. The findings can inform policy decisions and community programs. She has been using these photos collected by their Programme Working Groups to inform and influence decision makers in their community.

*So basically we are community led … we collect both quantitative and qualitative data. In terms of decision making we trying to focus more on photo-voicing which (this data) is more qualitative collected by Programs Working Groups*

**CBO representative-5, Advocacy officer**

##### Collaboration

Participants from the DOH and community-based organizations alluded to the fact that community level collaboration improve healthcare service delivery. Most collaborative activities include data sharing and analysis. While the DOH collected quantitative data, community-based organizations collected qualitative information. The use of multiple sources of data allowed for data triangulation. This increased data validity and also provided a more in-depth and holistic depiction of why interventions are failing or successful. One DOH representative said that data sharing helped with quality assurance.

A representative from a community organisation reiterated that data collected at the local level are usually large and complex. Through collaborative efforts with different stakeholders, they can rely on complementary expertise to analyse the data. Drawing lessons from PrEP information, she indicated that data sharing and analysis allowed for corrective action against inconsistencies the PrEP rollout programme, these anomalies could have been missed if the data was not shared and analysed by community based organisations.

*DOH didn’t realize that the provision of PrEP was inconsistent so when that PrEP rollout data was analysed they were surprised. The DOH realised that there were some patterns in the CLM data collected. This made them realised the need to collaborate with implementing partners and community based organisation who could assist in the data analysis.*

**CBO representative-6**

The DOH representative reiterated that data-sharing processes helped complement different stakeholder efforts.

*Remember we also have our own data (DOH District Health Information data in health facilities) and we mix (we analysed CLM data and DOH data). CLM data collection is complimentary to us (DOH data collected, and also they (Community based organisations) will do further analysis, and they also do a comparative analysis of almost 14 facilities*

**DOH representative-3**

#### Local Change

##### Programme Redesign

A CBO representative indicated that from the trend analysis conducted on PrEP data collected in the clinics showed the need to redesign the programme so as to increase knowledge, uptake linkages and retention on PrEP among priority populations.

*Based on the CLM findings, the PrEP program underwent a redesign to address the identified issues. For example, community engagement and education campaigns were initiated to raise awareness about PrEP and its benefits, targeting both high-risk individuals and the broader community.*

**CBO representative-7, Data Collector**

#### Immediate Outcomes

##### Rapid Response

The CBO representative said that she could see a positive direct link between data collection, collaboration, operational efficiency, and rapid response during COVID-19. She said that joint collaboration between the DOH and the City of Johannesburg led to an effective COVID-19 vaccine rollout plan being implemented.

*At some point during the rollout of COVID-19 vaccine the City ran out of vaccines but DOH still had vaccines. The DOH and City supported organisations partnered and supported each other in ensuring that there is access to the COVID-19 vaccines in the community. They also joined efforts in conducting community outreach activities.*

**CBO representative-9, Linkage Officer**

Another CBO representative said that their surveillance activities helped some health facilities to identify gaps and prioritize emerging critical issues.

*I was interviewing one of the Data Capturers who captures stats every month. When I asked about PrEP data they realised that some information was missing and they quickly notified the Facility Manager to help resolve the gaps that we had identified.*

**CBO representative-8, Data Collector**

#### Potential Impacts

Respondents also shed light on how important and relevant it has been for them to collect and use data at the community level has significantly improved their service delivery. One CBO data collector stated that:

*Community members possess local knowledge that may not be captured through traditional monitoring methods. CLM has helped us by ensuring we collect community level data that informs how we do our work.*

**CBO representative-10, Data Collector**

A health worker also highlighted that CLM enabled communities to identify and address their specific challenges. This can lead to the development of targeted and contextually relevant solutions, increasing the effectiveness of interventions and contributing to the overall sustainability of development efforts. A healthcare provider stated that:

*CLM helps communities figure out and solve their own problems. This can lead to creating solutions that really fit their situation, making actions more helpful and adding to the long-term success of development projects.*

**DOH representative-3**

## Discussion

This study sought to explore how data from CLM in the South African health system are perceived, utilized, and how these insights lead to positive outcomes. To create a thorough CLM data value chain that covers the entire process, from data collection to the potential influence of data on decision-making and program enhancements. Our findings revealed that CLM data collection played a crucial role in identifying internal gaps within the healthcare system. The analysis of CLM data also led to data validation, and the data facilitated improved program implementation, enhanced advocacy activities, and program redesign at the local level. We also found that collaboration at the community level was emphasized, through data sharing between the Department of Health (DOH) and community-based organizations. Finally, the findings suggest that CLM has the potential to lead to local changes. To our knowledge, this is the first study to comprehensively examine the perceptions of stakeholders involved in health programs in South Africa.

### The Significance of the CLM Data Value Chain Framework

The findings of this study revealed the fundamental role of the CLM Data Value Chain framework within the South African health system. Local data collection through the CLM model proved essential for surveillance and gap identification [3]. This is consistent with the literature, which emphasizes the importance of community engagement and local data collection for identifying context-specific challenges that program planners may not be aware of [1, 35]. By recognizing gaps within the healthcare system, CLM provided a robust foundation for program design advocacy and collaboration among stakeholders [23]. Tailoring interventions to the community’s unique needs enhances the program’s relevance and efficiency [36].

### Data Collection in Community-Led Monitoring (CLM)

In the realm of CLM within public health programs, this study’s findings shows the pivotal role of data collection in uncovering crucial gaps within healthcare systems. This aligns with existing literature emphasizing the value of local data collection for identifying contextual challenges and opportunities [37, 38]. Participants in this study, including community implementers and health workers, highlighted how CLM data collection helped to address critical issues such as gender-based violence (GBV) statistics in local clinics. These insights reinforce the notion that grassroots data collection empowers communities to take an active role in improving healthcare services [39, 40].

### Data Analysis and Validation Within the CLM Framework

Analysis of CLM data extends beyond mere data collection, serving as a powerful tool for data validation and issue identification. The participants highlighted how CLM data revealed trends and misconceptions related to healthcare practices. For instance, findings indicated an increase in teenage pregnancies, shedding light on the low uptake of contraceptives among teenagers. This resonates with the broader literature on data’s ability to unveil hidden patterns and trends [37, 38]. Additionally, CLM data analysis deepened the understanding of misconceptions surrounding HIV treatment, leading to more effective communication strategies. In essence, this study reveals that CLM data collection and analysis are essential components of a comprehensive approach to healthcare improvement, capable of driving positive change within local communities and advancing public health outcomes.

### Empowering Program Implementation and Advocacy

The study demonstrated that data collected through CLM significantly improved program implementation and advocacy efforts, aligning with previous research highlighting the efficiency of community-led activities in enhancing program performance [12, 35]. This empowering effect was also observed in studies emphasizing how community data drives implementation strategies and fosters trust between communities and health systems [12, 41]. CLM played a pivotal role in responding to health impacts, with interventions tailored to local needs and contexts [41]. CLM was instrumental in building community support for interventions, as seen in a study engaging teenage mothers in decision-making processes [42, 43].

### Quality Improvement and Collaborative Efforts

Data sharing and collaboration emerged as significant uses of CLM data, improving quality assurance and enhancing the relevance of collected data. This is in line with other studies, for instance Carr and Littler in their work on sharing research data to improve public health demonstrated the importance of data sharing among stakeholders [44]. The study highlighted the importance of data triangulation using multiple sources, which leads to more effective interventions and better health outcomes. This aligns with studies that emphasize the value of collaborative activities for stakeholders to learn from each other, drawing insights from the data [45, 46]. By working together, researchers, policymakers, and community members pool their expertise and perspectives, leading to comprehensive understandings of the issues being studied [5, 45].

### Influence of Community-Led Monitoring on Healthcare Program Redesign

Incorporating the insights from CLM data, the study reveals the significant influence of CLM on health program design and redesign. The data analysis, informed by CLM, uncovered critical trends related to PrEP usage in clinics, prompting the need for program modifications. In response, the PrEP program underwent a strategic transformation, including community engagement and educational campaigns aimed at increasing awareness and uptake of PrEP among priority populations. This demonstrates how CLM not only identifies issues but also acts as a catalyst for adaptive and responsive program redesign, aligning with the broader literature on data-driven decision-making and the importance of community engagement in public health interventions [47, 48].

### Immediate Outcomes and Rapid Response in CLM

Within the CLM framework, immediate outcomes are observed, demonstrating the practical impact of this approach. Rapid response mechanisms, driven by data collection and collaboration, play a crucial role, particularly during crisis situations like the COVID-19 pandemic. Collaboration between the Department of Health (DOH) and the City of Johannesburg, informed by CLM data, led to an efficient COVID-19 vaccine rollout plan. Notably, when vaccine shortages occurred, joint efforts ensured community access to vaccines and supported community outreach initiatives. In summary, CLM demonstrates its immediate impact through rapid response mechanisms and improved surveillance, contributing to better health outcomes.

### Fostering Accountability and Sustainable Community Engagement

CLM promotes accountability, trust, and transparency in the health system, improving access to health services [46, 49]. By empowering communities to be active participants in surveillance, data reporting becomes more comprehensive and accurate [50, 51]. This inclusivity in decision-making processes ensures that interventions are tailored to the community’s unique needs, building sustainable, efficient and effective program implementation [41, 52].

This study explored the transformative impact of the CLM Data Value Chain framework in the South African health system. The seamless progression from data collection to tailored program implementation, advocacy, and collaboration significantly impacted healthcare outcomes. CLM emerged as a powerful tool for building sustainable, efficient and effective program implementation. The findings emphasize the importance of embracing the CLM model to address community-specific challenges effectively and drive sustainable health improvements.

### Strength and Limitation of the Study

The strengths of this study was in ability to conduct an in-depth exploration of the research question. Through this, it was possible to acquire rich data and gain insights from the experiences and perspectives of the participants to reach a thorough understanding of CLM and how stakeholders viewed and used CLM data. The flexibility of the research process allowed for the data collection to be adapted to the various scenarios and settings.

The limitations includes; biases, and preconceptions which could have influenced the data collection, analysis, and interpretation of the findings. Generally, the lack of standardization in the qualitative research process and methodology makes it difficult to compare these findings with other studies.

### Conclusion

CLM has been identified as a powerful tool for improving program implementation, facilitating redesign and ultimately the uptake of quality health services. The involvement of communities in data collection and analysis enables stakeholders to learn from each other, draw lessons from the data, and improve the quality and relevance of the collected data. CLM also promotes accountability and transparency in the health system, and improves access to quality health services. These are essential components of successful surveillance and intervention programs in the public health sector. These approaches can improve the accuracy and completeness of surveillance data, increase the effectiveness of interventions, and build trust between public health systems and communities. Collaboration between stakeholders is a key success factor for CLM because it enables mutual interdependence among stakeholders. Furthermore, data sharing and collaboration can help ensure that data is collected based on the needs and priorities of different stakeholders.

It is important to note that community engagement requires careful planning and resources allocation to be successful. This involves building the capacity within communities to participate effectively in surveillance and intervention programs. Additionally, transparency and clear communication between the public health system and communities are critical to ensuring that the community understands the purpose and goals of monitoring activities. It is, therefore, crucial that public health systems invest in community engagements and CLM to improve the overall health of communities.
